# Dexamethasone and lidocaine suppress eosinophilopoiesis from umbilical cord blood cells

**DOI:** 10.1186/s12948-020-00138-1

**Published:** 2020-12-02

**Authors:** Masato Muraki, Hirohito Kita, Gerald J. Gleich

**Affiliations:** 1 Division of Allergic Diseases and Department of Medicine, Mayo Clinic, Rochester, MN USA; 2 Department of Respiratory Medicine and Allergology, Kindai University Nara Hospital, 1248-1 Otoda-cho, Ikoma, Nara, 630-0293 Japan; 3 Division of Allergy, Asthma, and Clinical Immunology, Department of Medicine, Mayo Clinic, Scottsdale, AZ USA; 4 Department of Dermatology, University of Utah Health Sciences Center, Salt Lake City, Utah USA

**Keywords:** Dexamethasone, Lidocaine, Eosinophilopoiesis, Eosinophil-derived neurotoxin, Eosinophil peroxidase, Umbilical cord blood

## Abstract

**Background:**

Eosinophils play an important role in allergic inflammation. Glucocorticosteroids have been used as an anti-inflammatory medication for inflammatory diseases involving eosinophil infiltration. Some effect of nebulized lidocaine has been reported when treating certain patients with asthma, which is also an inflammatory disease. The goal of this study was to examine the effects of dexamethasone and lidocaine on eosinophil proliferation and differentiation using a model of human umbilical cord blood mononuclear cells (UCMC) cultured with IL-5.

**Methods:**

UCMC were cultured with IL-5 (5 ng/mL) for 4 weeks. The effects of dexamethasone and lidocaine on the number and morphology of eosinophilic cells were visualized with Wright-Giemsa and cyanide-resistant peroxidase stains. Moreover, the effect on eosinophil-derived neurotoxin (EDN) and eosinophil peroxidase (EPX) contents in cultured cells were evaluated using radioimmunoassay.

**Results:**

The number of eosinophilic cells and EDN and EPX content in cultured cells increased in a time-dependent manner in the presence of IL-5. Dexamethasone treatment slightly decreased the number of eosinophilic cells in one week, but this effect was lost in 2–4 weeks. Macrophages in cultured UCMC treated with dexamethasone contained more eosinophil granule proteins. Both EDN and EPX content in cultured cells were reduced by dexamethasone. Lidocaine decreased the number of eosinophilic cells and reduced both EDN and EPX contents in cultured cells.

**Conclusions:**

Dexamethasone suppressed the production of eosinophil granule proteins and may also induce apoptosis of eosinophils, while lidocaine suppresses eosinophilopoiesis.

## Background

Eosinophils play an important role in allergic diseases, including asthma. Corticosteroid therapy is effective for treating eosinophilic diseases [1–4]. However, the mechanisms of eosinophil development and survival are only partially understood [5]. Both the inhibitory [6] and stimulatory [7] effects of glucocorticoids are reported; however, their effect on eosinophilopoiesis is still unclear.

Inhaled or systemic corticosteroids are ineffective treatments in many patients with severe asthma, and few treatment options exist for patients with steroid-resistant asthma [8], although inhaled corticosteroids are the initial controllers administered for treating asthma [9].

IL-5 is the main cytokine targeting eosinophilopoiesis [10], and the therapeutic efficacy of humanized monoclonal antibodies that target IL-5 and IL-5 receptor α for severe asthma has been established [11, 12]. However, these biomedicines incur high medical costs. Some studies proved that nebulized lidocaine is a useful therapy for control of asthma, even steroid-dependent asthma [13, 14]. Lidocaine and its analogue also affect the activity and survival of mature eosinophils [15] and inhibit allergen-induced eosinophilic inflammation [16–18]. Conversely, some studies report that they do not affect airway eosinophilia [19, 20]. Thus, there is no definitive answer to the nature of the effect of steroids on eosinophilopoiesis and no direct study on the effect of lidocaine on eosinophilopoiesis.

In the present study, the effect of dexamethasone and lidocaine on eosinophilopoiesis stimulated by IL-5 was investigated. Eosinophilopoiesis was evaluated as the number and morphology of eosinophilic cells as well as the eosinophil granule proteins obtained from human umbilical cord blood mononuclear cells (UCMC) culture.

## Methods

### Isolation of UCMC

Human umbilical cord blood was collected during normal deliveries of new-born infants at the Mayo Clinic, Methodist Campus. Heparin-treated umbilical cord blood from normal individuals were immediately layered over Histopaque-1077 (Sigma-Aldrich, St. Louis, MO), and the tubes containing the cord blood were centrifuged at 400×*g* for 30 min at room temperature to obtain a mononuclear cell fraction. After washing twice with phosphate-buffered saline (PBS) and 1% bovine calf serum (HyClone Laboratories Inc., Logan, UT), UCMC were suspended in RPMI-1640 medium (Celox Laboratories Inc., Cherry Hill, NJ) supplemented with 10% bovine calf serum, 2 mM L-glutamine (Sigma-Aldrich, St. Louis, MO), and 50 µg/mL gentamicin (pH 7.4; Sigma-Aldrich, St. Louis, MO). A part of the suspended cells was counted on haemocytometer using Randolph's stain. Isolated UCMC were washed and 2 × 10^6^ cells/mL was resuspended in the medium.

### Cell culture

Flat-bottomed 96-well cell culture plates (Corning Inc., Corning, NY) were coated with 100 mg/mL of hyaluronic acid (HA) in PBS (Sigma-Aldrich, St. Louis, MO) at 37 °C for 3 h. UCMC (2 × 10^5^ cells) were suspended in 200 µL of medium and cultured in the presence of 5 ng/mL of IL-5 (a gift from Schering-Plough Research Institute, Kenilworth, NJ) or medium alone at 37 °C in 5% CO_2_. The cells suspended in the presence of IL-5 were also cultured with 1 × 10^–9^ M–1 × 10^–6^ M of dexamethasone (Sigma-Aldrich, St. Louis, MO), 3 × 10^–5^ M–1 × 10^–3^ M of lidocaine (Sigma-Aldrich, St. Louis, MO) or medium alone. Half the volume of culture medium was changed weekly. Four wells were utilized for evaluating the cell harvest, morphology, and granule proteins. All assays were carried out in duplicate.

### Harvest and morphology of cultured cells

Viability and total number of cultured cells were determined by trypan blue exclusion, and the cells developed in the culture were characterized using Wright-Giemsa stain and cyanide-resistant peroxidase stain. The number of total cells and the cell components were counted weekly for 4 weeks.

### Eosinophil granule protein content in cultured cells

To examine eosinophil differentiation, we analyse the contents of eosinophil granule proteins, including eosinophil-derived neurotoxin (EDN) and eosinophil peroxidase (EPX), in the lysates of cultured cells. After half the volume (100 µL) of medium supernatant was carefully removed from each well, 100 µL of 1% NP-40 (Sigma-Aldrich, St. Louis, MO)/0.01 N HCl was poured into the wells. The cell lysate was stored at − 20 °C until measurement of EDN and EPX.

The concentration of EDN in sample lysate was measured by radioimmunoassay (RIA). The RIA for EDN is a double-antibody competition assay in which radioiodinated EDN, rabbit anti-EDN antibody, and burro anti-rabbit IgG are used as described previously [21, 22].

The concentration of EPX in sample lysate was measured by RIA [23], which was modified as follows. Before the assay, Immulon-4 96-well plates (Dynex Technologies Inc., Chantilly, VA) were coated overnight at 4 °C with 100 µL of anti-human EPO antibody (5 µg/mL in PBS) and blocked with 200 µL of phosphate, protamine, foetal bovine serum, and EDTA (PPF-E) for 2 h at room temperature. After washing the wells with washing buffer (0.1 M PO_4_, pH 7.5; Tween 20, 10 mL/L), 100 µL of samples diluted with PPF-E or standard control (purified EPX from sera of hypereosinophilic syndrome patients) were added to the wells in duplicate and incubated overnight at 4 °C. Next, wells were washed again with washing buffer. A second antibody, anti-human EPX antibody radiolabelled with I^125^, was added to the wells (50 ng/mL in PPF-E buffer, 100 µL/well) and incubated for 6 h at room temperature. The wells were then washed, and antibodies radiolabelled with I^125^ were counted in a gamma scintillation counter.

### Statistical analysis

Data were represented as mean ± SE, and the statistical significance of the differences was assessed with paired nonparametric Wilcoxon signed-rank test.

## Results

### Kinetic changes in eosinophilic cells

**Fig. 1:**
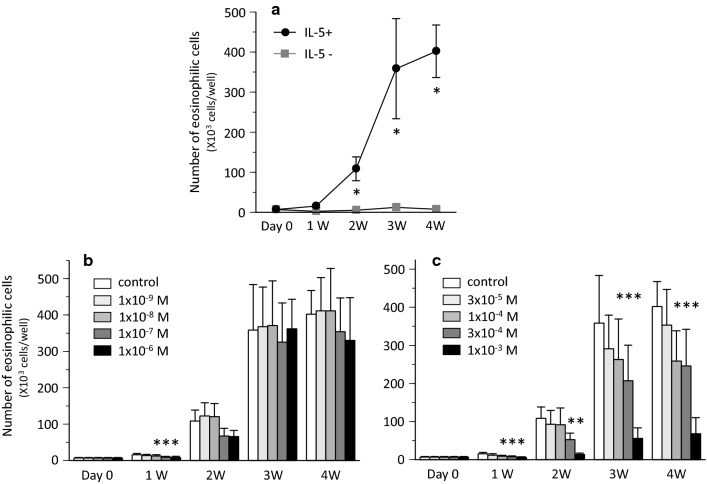
Harvest of eosinophilic cells cultured from UCMC over study period. **a** Control stimulated with or without IL-5 (* *P* < 0.05 vs. IL-5-). **b** Effect of dexamethasone on eosinophilopoiesis (* *P* < 0.05 vs. control with IL-5). **c** Effect of lidocaine on eosinophilopoiesis (* *P* < 0.05 vs. control with IL-5)

The total cultured cell number decreased temporally after one week when UCMCs were cultured with IL-5. During and after 2 weeks, the total UCMC number increased. The number of eosinophilic cells at each week is shown in Fig. 1a. No eosinophilic cells were detectable on day 0, but the number of eosinophilic cells increased time-dependently in the presence of IL-5. After 4 weeks, 96.9% of the total cells harvested were eosinophilic cells. In contrast, in the absence of IL-5, no eosinophilic cells were detectable after 4 weeks. The treatment of the cultured cells with dexamethasone dose-dependently suppressed eosinophilopoiesis stimulated with IL-5 after one week. However, there were no significant differences between control and dexamethasone-treated groups during and after 2 weeks (Fig. 1b). In comparison, lidocaine dose-dependently suppressed eosinophilopoiesis during and after one week (Fig. 1c). Conversely, the number of macrophages in the group treated with lidocaine increased more dose-dependently than the number in control group (Additional file 1).

### Morphological findings of cultured cells

**Fig. 2:**
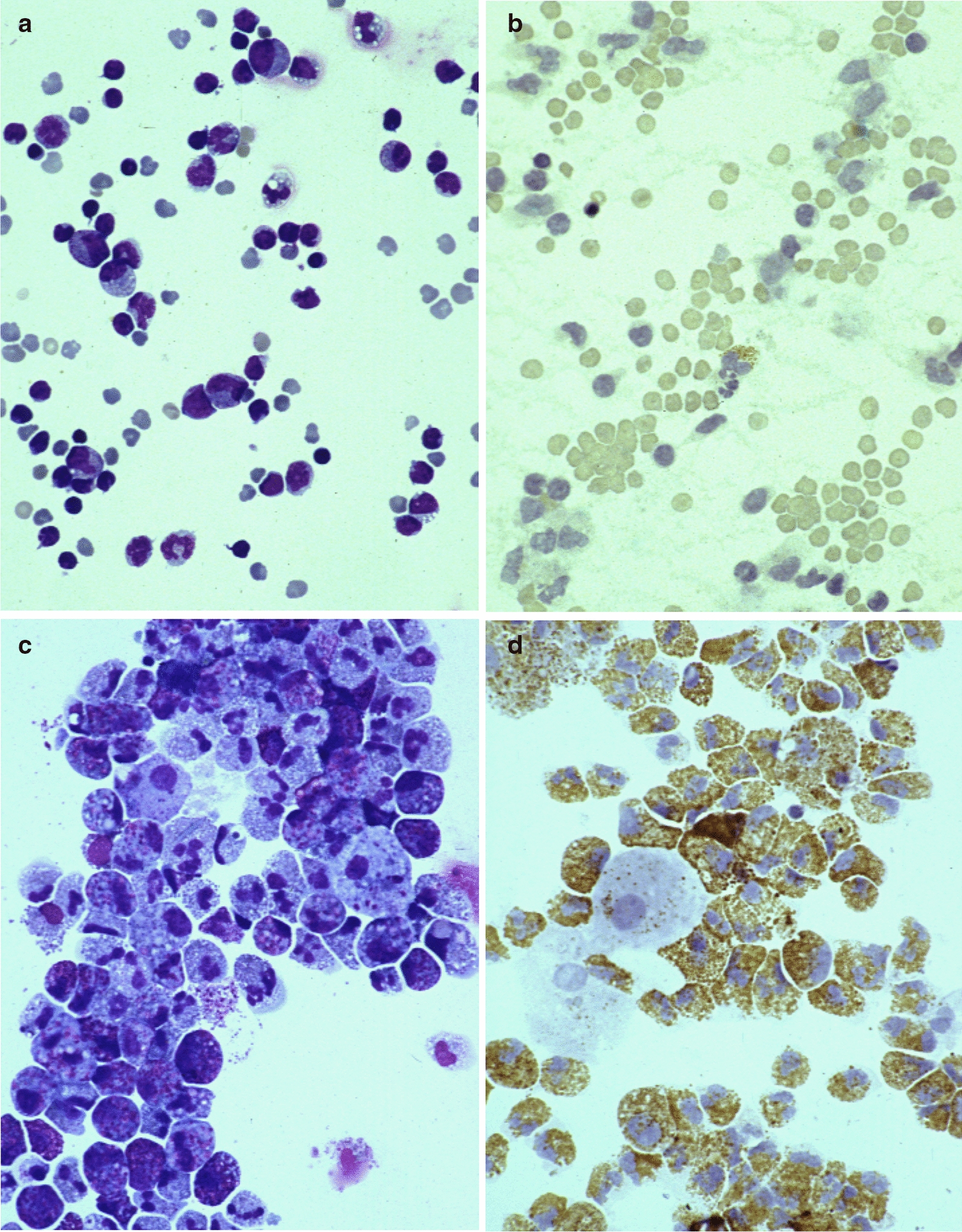

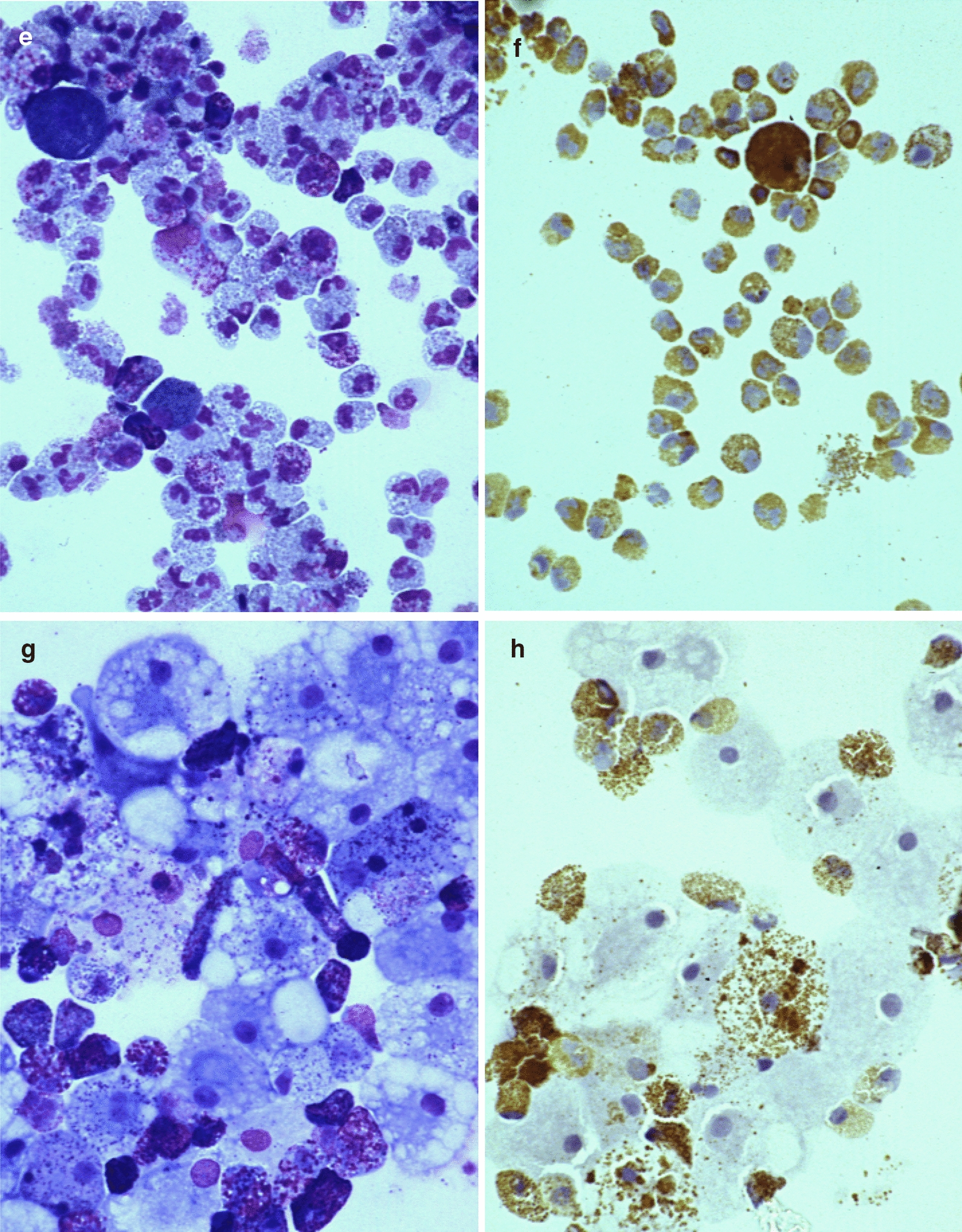
Microscopic findings of cultured cells stained by Wright-Giemsa, × 160 (left) and cyanide-resistant peroxidase, × 160 (right). **a**, **b** Isolated UCMC on day 0. **c**, **d** Cultured cells in control stimulated with IL-5 after 4 weeks. **e**, **f** Cultured cells treated with 1 × 10^–6^ M dexamethasone after 4 weeks. **g**, **h** Cultured cells treated with 1 × 10^–3^ M lidocaine after 4 weeks

To examine the morphology of cultured cells, we stained them with Wright-Giemsa stain and cyanide-resistant peroxidase. Cyanide-resistant peroxidase stain specifically detects EPX. On the first day, a majority of UCMCs were mononuclear cells, including lymphocytes, monocytes or undifferentiated cells, and erythrocytes. Only a small percentage (3.5 ± 0.7%) of cells stained positive with cyanide-resistant peroxidase (Fig. 2a, b).

When cultured with IL-5, most (96.9%) of the cultured cells showed myeloid cell or granulocyte morphology that stained positive with cyanide-resistant peroxidase after 4 weeks (Fig. 2c, d). Macrophages were observed in 2.6% of the cultured cells and slightly phagocytosed eosinophil granules (Fig. 2c, d).

When UCMC were cultured with IL-5 in the presence of 10^–6^ M dexamethasone, most (96.7%) of the cultured cells were eosinophilic cells, and 2.6% of the cells observed in the 4th week were macrophages (Fig. 2e, f). However, macrophages in cultured cells treated with dexamethasone phagocytosed eosinophilic granules much more than in the absence of dexamethasone (Fig. 2e, f). In cultured cells treated with 10^–3^ M lidocaine, 55.9% were eosinophilic cells and 43.9% were macrophages at 4 weeks (Fig. 2g, h). Eosinophilic cells treated with lidocaine stained by Wright-Giemsa seemed to be stained more intensely than control eosinophilic cells (Fig. 2g). A part of the macrophages phagocytosed eosinophilic granules (Fig. 2g, h).

### EDN and EPX content in cultured cells

**Fig. 3:**
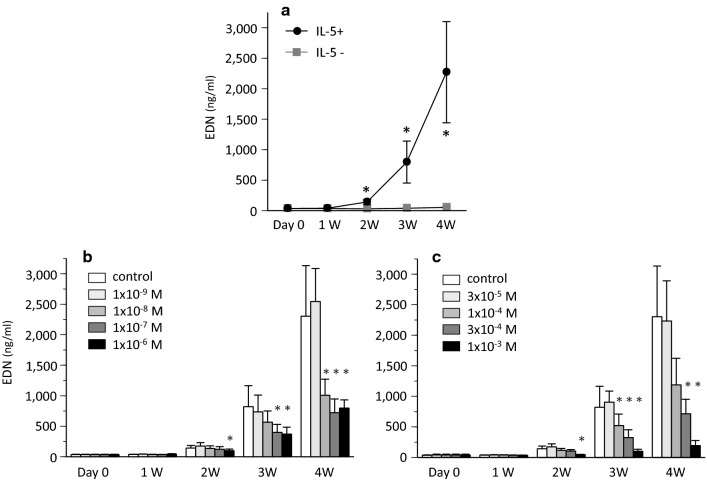
EDN content in cultured cells over study period. **a** EDN levels in cell lysate of control stimulated with or without IL-5 (* *P* < 0.05 vs IL-5-). **b** Effect of dexamethasone on EDN content (* *P* < 0.05 vs control with IL-5). **c** Effect of lidocaine on EDN content (* *P* < 0.05 vs control with IL-5)

**Fig. 4:**
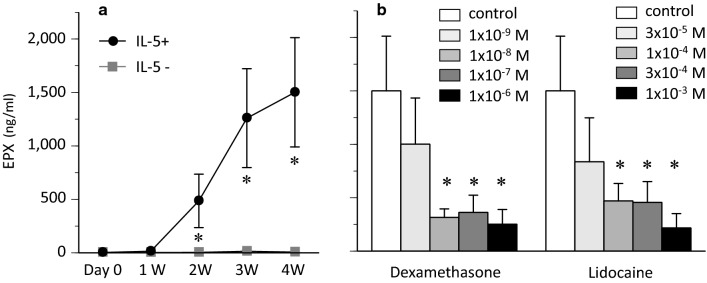
EPX content in cultured cells. **a** EPX levels in cell lysate of control stimulated with or without IL-5 over study period (* *P* < 0.05 vs IL-5-). **b** Effect of dexamethasone or lidocaine on EPX content in cultured cells after 4 weeks (* *P* < 0.05 vs control with IL-5)

To analyse the maturation of eosinophils from UCMCs, the concentration of EDN and EPX in cell lysate was measured. The EDN and EPX content in cultured cells with IL-5 are shown in Figs. 3 and 4. EDN and EPX content in control cultured cells increased in a time-dependent manner (Figs. 3a and 4a).

During and after 2 weeks, the treatment with dexamethasone dose-dependently reduced EDN content despite no significant differences in the number of eosinophils between control and dexamethasone-treated groups (Fig. 3b). The treatment with dexamethasone also reduced EPX content after 4 weeks (Fig. 4b).

Lidocaine dose-dependently reduced EDN content during and after 2 weeks (Fig. 3c). EPX content was also dose-dependently reduced after 4 weeks (Fig. 4b).

**Fig. 5:**
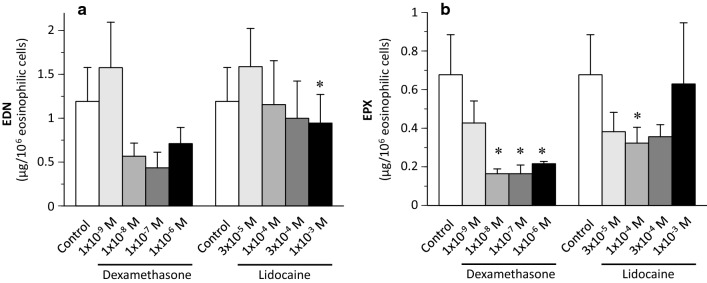
Effect of dexamethasone or lidocaine on EDN and EPX levels per one million eosinophilic cells after 4 weeks. **a** EDN levels (* *P* < 0.05 vs control with IL-5). **b** EPX levels (* *P* < 0.05 vs control with IL-5)

Finally, we calculated the EDN or EPX levels and normalized them to the cell number (1 × 10^6^ cells) after 4 weeks. EDN levels for one million eosinophils treated with dexamethasone or lidocaine decreased, although with partially or no significant differences (Fig. 5a). EPX levels per million eosinophilic cells treated with dexamethasone or lidocaine also decreased after 4 weeks (Fig. 5b).

## Discussion

Eosinophils play an important role in the pathogenesis of many diseases, especially those affecting the airway, skin, or digestive tract. Biological preparations have been developed that directly or indirectly target eosinophils in asthma [24]. Eosinophil regulation is crucial in allergic diseases. Umbilical cord blood contains many progenitors to various kinds of blood cells. Several studies have shown that cultured eosinophils can be generated from human UCMC in the presence of IL-5, IL-3, and granulocyte–macrophage colony-stimulating factor (GM-CSF) [25, 26]. HA also enhances proliferation during eosinophilopoiesis from UCMC [27, 28]. In this study, we used IL-5 with HA-coated flask for UCMC culture, because GM-CSF and IL-3 non-specifically stimulate all inflammatory cells except eosinophils [12, 29] or down-regulate IL-5 receptor α [30].

IL-5 appears to be the critical cytokine specific to eosinophil development which mechanistically acts to drive expansion and survival of mature eosinophils [31]. Patients with severe eosinophilic asthma have an exaggerated eosinophilopoietic process in their airways. Targeting IL-5-driven eosinophil differentiation locally within the lung maybe of relevance for optimal control of airway eosinophilia and asthma [32]. Inhaled corticosteroids are initially administered as controller medication for asthma, and biological preparations targeting IL-5/IL-5 receptor are used for some populations with corticosteroid-resistant asthma [11]. Nebulized lidocaine, however, are reportedly effective in animal [16, 33] and human [13, 14] studies, and could potentially replace expensive biomedicines, if used as an inhalant. In this study, we examined the effects of corticosteroid and lidocaine on eosinophil production under IL-5 stimulation.

In the present study, eosinophils had differentiated and proliferated weekly on IL-5 stimulation. Dexamethasone slightly suppressed eosinophil production after one week but did not significantly affect the production during and after 2 weeks, in contrast to the findings of previous reports [5–7]. However, dexamethasone reduced EDN content in cultured cells. Corticosteroids show the eosinophil apoptosis-promoting effect and enhance the capacity of monocyte-derived macrophages to phagocytose apoptotic eosinophils [34]. This study also showed that macrophages treated with dexamethasone included eosinophil granule proteins in cytoplasm much more than macrophages in control, suggesting that apoptosis might be induced, and that phagocytosis is enhanced. Despite this effect on phagocytosis, there were no statistically significant differences between produced eosinophil numbers. This may indicate that dexamethasone delays eosinophil maturation.

Lidocaine inhibits cytokine-mediated eosinophil survival and hastened the apoptosis of eosinophils delayed by IL-5 [15, 35]. In the present study, lidocaine, unlike dexamethasone, suppressed eosinophil production from when the cells were initially cultured. Morphologically, granules in eosinophilic cells treated with lidocaine seemed to be more intensely stained than in control eosinophilic cells on Wright-Giemsa staining. Lidocaine also reduced EDN and EPX content in cultured cells stimulated with IL-5. Therefore, it was suggested that lidocaine might inhibit eosinophil differentiation, proliferation, or maturation.

Infiltration of eosinophils into the airway from the bone marrow and blood is the hallmark of eosinophilic asthma [36]. Airway eosinophilia in asthmatic patients can also arise by in situ differentiation and is driven by the locally elaborated eosinophilopoietic cytokine IL-5 [37]. ILC2 cells can promote the persistence of airway eosinophilia in patients with severe asthma through uncontrolled localized production of type 2 cytokines such as IL-5 [37]. Dexamethasone does not inhibit in situ eosinophil differentiation and proliferation but may induce inhibition of eosinophil maturation and acceleration of apoptosis as indicated by the result of this study. Local therapy of lidocaine may inhibit eosinophilopoiesis in the airway. Therefore, lidocaine may represent a new class of therapeutic agents to treat patients with eosinophilic airway diseases and may be useful even in steroid-resistant asthma. The combination of lidocaine and corticosteroid may show synergistic effect by different mechanisms as the effects of these agents on eosinophil maturation appear to be different.

The limitation of the study is that an accurate measurement of eosinophil granule protein content in pure eosinophilic cells was impossible in the present system, due to contamination of eosinophil-phagocytic macrophages. Therefore, macrophages treated with high concentrations of dexamethasone or lidocaine phagocytosed more eosinophils, and the EDN or EPX levels per normalized eosinophil number in this study were considered to be high, which is contrary to reality. Although there were differences between the EDN levels and EPX levels per normalized eosinophil number treated with dexamethasone, it was unclear whether dexamethasone specifically suppressed EPX production (Fig. 5). Furthermore, although the maturation of eosinophils was evaluated as EPX and EDN content in cultured cells alternatively in this study, the maturation was not directly measured, and it was thought that more sensitive methods such as comparison of the expression of eotaxin receptor CCR3 (CD193) [38] should have been used.

## Conclusions

Our findings showed that dexamethasone had no significant effect on the final number of eosinophil production after 4 weeks and suppressed the amount of granule proteins, while lidocaine suppressed both the number of eosinophils and granule protein levels. In addition, high concentrations of dexamethasone or lidocaine seemed to morphologically induce eosinophil phagocytosis by macrophages. Thus, dexamethasone may inhibit eosinophil maturation and induce apoptosis. Lidocaine may inhibit eosinophil differentiation, proliferation, or maturation stimulated by IL-5. Furthermore, not only corticosteroids, but also lidocaine are important medications in eosinophilic inflammation by different mechanisms. The different effects of corticosteroids and lidocaine on eosinophilopoiesis in this study are expected to play a role in treating steroid-resistant eosinophilic inflammatory diseases in future. Further study is needed to establish this therapeutic role.

## Supplementary information

**Additional file 1.** Effect of dexamethasone or lidocaine on the number of macrophages cultured from UCMC after 4 weeks. (* *P* < 0.05 vs control with IL-5).

## Data Availability

The datasets used and/or analyzed during the current study are available from the corresponding author on reasonable request.

## Abbreviations

UCMC: Umbilical cord blood mononuclear cells
EDN: Eosinophil-derived neurotoxin
EPX: Eosinophil peroxidase
PBS: Phosphate-buffered saline
HA: Hyaluronic acid
RIA: Radioimmunoassay
PPF-E: Phosphate, protamine, foetal bovine serum, and EDTA

## References

[1] McDowell PJ, Heaney LG (2020). Different endotypes and phenotypes drive the heterogeneity in severe asthma. Allergy.

[2] Suzuki Y, Suda T (2019). Eosinophilic pneumonia: a review of the previous literature, causes, diagnosis, and management. Allergol Int.

[3] Steinbach EC, Hernandez M, Dellon ES (2018). Eosinophilic esophagitis and the eosinophilic gastrointestinal diseases: approach to diagnosis and management. J Allergy Clin Immunol Pract.

[4] Ramsahai JM, Wark PA (2018). Appropriate use of oral corticosteroids for severe asthma. Med J Aust.

[5] Willebrand R, Voehringer D (2017). Regulation of eosinophil development and survival. Curr Opin Hematol.

[6] Bjornson BH, Harvey JM, Rose L (1985). Differential effect of hydrocortisone on eosinophil and neutrophil proliferation. J Clin Invest.

[7] Barr RD, Volaric Z, Koekebakker M (1987). Stimulation of human eosinophilopoiesis by hydrocortisone in vitro. Acta Haematol.

[8] Peters MC, Wenzel SE (2020). Intersection of biology and therapeutics: type 2 targeted therapeutics for adult asthma. Lancet.

[9] Global Initiative for Asthma. The global strategy for asthma management and prevention updated 2020. http://www.ginasthma.org. Accessed 25 June 2020.

[10] Johansson K, Malmhäll C, Ramos-Ramírez P, Rådinger M (2018). Bone marrow type 2 innate lymphoid cells: a local source of interleukin-5 in interleukin-33-driven eosinophilia. Immunology.

[11] Nagase H, Ueki S, Fujieda S (2020). The roles of IL-5 and anti-IL-5 treatment in eosinophilic diseases: Asthma, eosinophilic granulomatosis with polyangiitis, and eosinophilic chronic rhinosinusitis. Allergol Int.

[12] Dougan M, Dranoff G, Dougan SK (2019). GM-CSF, IL-3, and IL-5 Family of Cytokines: regulators of inflammation. Immunity.

[13] Decco ML, Neeno TA, Hunt LW, O'Connell EJ, Yunginger JW, Sachs MI (1999). Nebulized lidocaine in the treatment of severe asthma in children: a pilot study. Ann Allergy Asthma Immunol.

[14] Hunt LW, Swedlund HA, Gleich GJ (1996). Effect of nebulized lidocaine on severe glucocorticoid-dependent asthma. Mayo Clin Proc.

[15] Okada S, Hagan JB, Kato M, Bankers-Fulbright JL, Hunt LW, Gleich GJ (1998). Lidocaine and its analogues inhibit IL-5-mediated survival and activation of human eosinophils. J Immunol.

[16] Muraki M, Iwanaga T, Haraguchi R, Kubo H, Tohda Y (2008). Continued inhalation of lidocaine suppresses antigen-induced airway hyperreactivity and airway inflammation in ovalbumin-sensitized guinea pigs. Int Immunopharmacol.

[17] Serra MF, Neves JS, Couto GC, Cotias AC, Pão CR, Olsen PC (2016). JM25-1, a lidocaine analog combining airway relaxant and antiinflammatory properties: implications for new bronchospasm therapy. Anesthesiology.

[18] Kim HS, Won S, Lee EK, Chun YH, Yoon JS, Kim JT (2017). Effect of proparacaine in a mouse model of allergic rhinitis. Clin Exp Otorhinolaryngol.

[19] Duong M, Wilson AM, Jayaram L, Dolovich M, Hargreave F (2008). The effect of inhaled lidocaine-hydrofluoroalkane 134a in prednisone-dependent eosinophilic bronchitis. Eur Respir J.

[20] Nafe LA, Guntur VP, Dodam JR, Lee-Fowler TM, Cohn LA, Reinero CR (2013). Nebulized lidocaine blunts airway hyper-responsiveness in experimental feline asthma. J Feline Med Surg.

[21] Plager DA, Henke SA, Matsuwaki Y, Madaan A, Squillace DL, Dierkhising RA (2009). Pimecrolimus reduces eosinophil activation associated with calcium mobilization. Int Arch Allergy Immunol.

[22] Muraki M, Gleich GJ, Kita H (2011). Antigen-specific IgG and IgA, but not IgE, activate the effector functions of eosinophils in the presence of antigen. Int Arch Allergy Immunol.

[23] Abu-Ghazaleh RI, Dunnette SL, Loegering DA, Checkel JL, Kita H, Thomas LL (1992). Eosinophil granule proteins in peripheral blood granulocytes. J Leukoc Biol.

[24] O'Sullivan JA, Bochner BS (2018). Eosinophils and eosinophil-associated diseases: an update. J Allergy Clin Immunol.

[25] Shalit M, Sekhsaria S, Malech HL (1995). Modulation of growth and differentiation of eosinophils from human peripheral blood CD34+ cells by IL5 and other growth factors. Cell Immunol.

[26] Walsh GM, Hartnell A, Moqbel R, Cromwell O, Nagy L, Bradley B (1990). Receptor expression and functional status of cultured human eosinophils derived from umbilical cord blood mononuclear cells. Blood.

[27] Ohashi H, Takei M, Ide Y, Ishii H, Kita H, Gleich GJ (1999). Effect of interleukin-3, interleukin-5 and hyaluronic acid on cultured eosinophils derived from human umbilical cord blood mononuclear cells. Int Arch Allergy Immunol.

[28] Hamann KJ, Dowling TL, Neeley SP, Grant JA, Leff AR (1995). Hyaluronic acid enhances cell proliferation during eosinopoiesis through the CD44 surface antigen. J Immunol.

[29] Becher B, Tugues S, Greter M (2016). GM-CSF: from growth factor to central mediator of tissue inflammation. Immunity.

[30] Gauvreau GM, Ellis AK, Denburg JA (2009). Haemopoietic processes in allergic disease: eosinophil/basophil development. Clin Exp Allergy.

[31] Johnston LK, Hsu CL, Krier-Burris RA, Chhiba KD, Chien KB, McKenzie A (2016). IL-33 precedes IL-5 in regulating eosinophil commitment and is required for eosinophil homeostasis. J Immunol.

[32] Sehmi R, Smith SG, Kjarsgaard M, Radford K, Boulet LP, Lemiere C (2016). Role of local eosinophilopoietic processes in the development of airway eosinophilia in prednisone-dependent severe asthma. Clin Exp Allergy.

[33] Wang L, Wang M, Li S, Wu H, Shen Q, Zhang S (2018). Nebulized lidocaine ameliorates allergic airway inflammation via downregulation of TLR2. Mol Immunol.

[34] Ilmarinen P, Kankaanranta H (2014). Eosinophil apoptosis as a therapeutic target in allergic asthma. Basic Clin Pharmacol Toxicol.

[35] Ohnishi T, Kita H, Mayeno AN, Okada S, Sur S, Broide DH (1996). Lidocaine in bronchoalveolar lavage fluid (BALF) is an inhibitor of eosinophil-active cytokines. Clin Exp Immunol.

[36] Yi S, Zhai J, Niu R, Zhu G, Wang M, Liu J (2018). Eosinophil recruitment is dynamically regulated by interplay among lung dendritic cell subsets after allergen challenge. Nat Commun.

[37] Smith SG, Chen R, Kjarsgaard M, Huang C, Oliveria JP, O'Byrne PM (2016). Increased numbers of activated group 2 innate lymphoid cells in the airways of patients with severe asthma and persistent airway eosinophilia. J Allergy Clin Immunol.

[38] Hassani M, van Staveren S, van Grinsven E, Bartels M, Tesselaar K, Leijte G (2020). Characterization of the phenotype of human eosinophils and their progenitors in the bone marrow of healthy individuals. Haematologica.

