# Supplementary material for: Dexamethasone and lidocaine suppress eosinophilopoiesis from umbilical cord blood cells
Source: Clin Mol Allergy. 2020 Dec 2;18:24. doi: 10.1186/s12948-020-00138-1 (PMC7713138; doi:10.1186/s12948-020-00138-1)
Supplement: Supplementary file 1 — Additional file 1. Effect of dexamethasone or lidocaine on the number of macrophages cultured from UCMC after 4 weeks. (* P < 0.05 vs control with IL-5). [file 12948_2020_138_MOESM1_ESM.pptx]

## Slide 1
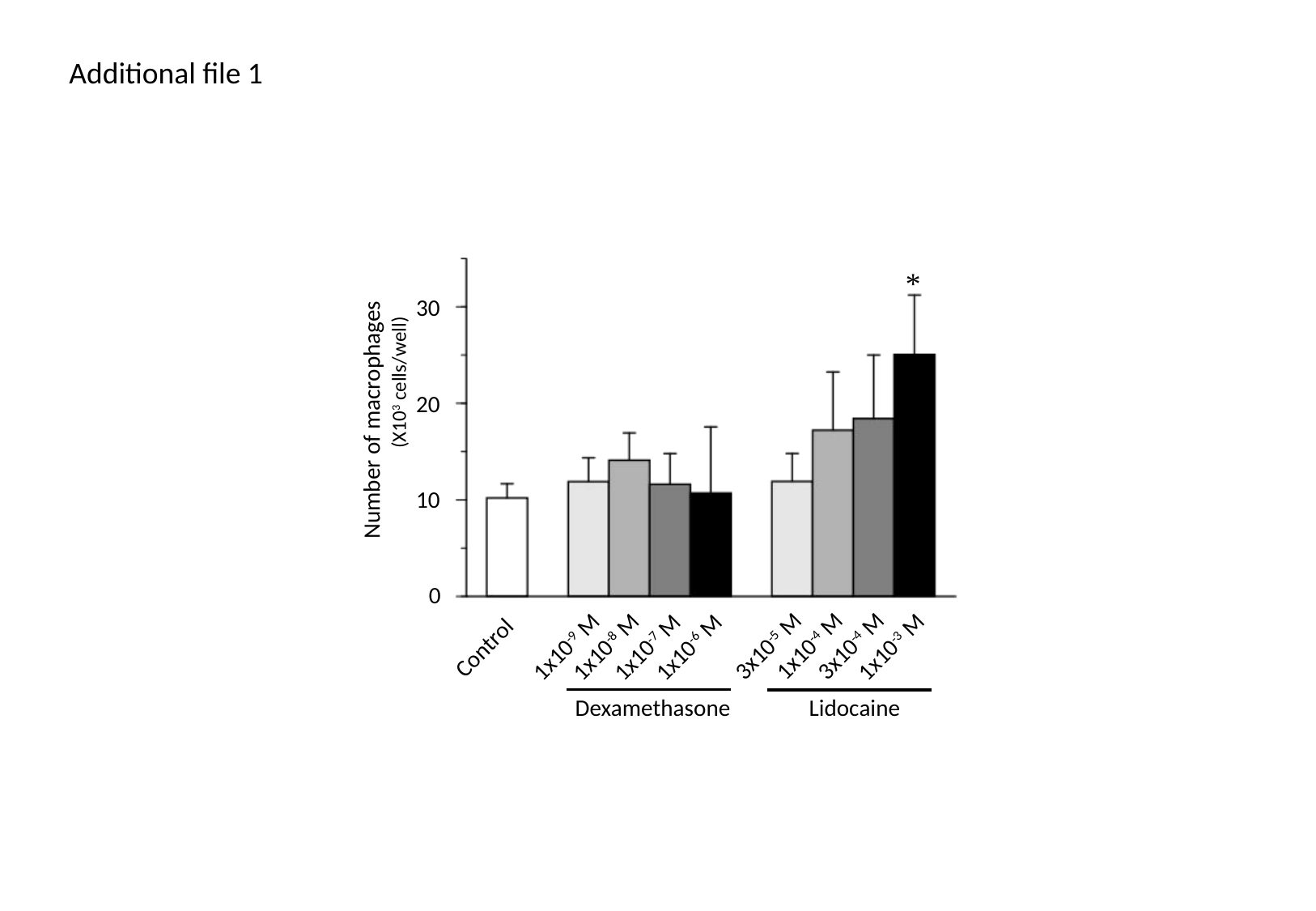

Additional file 1
*
30
20
10
0
(X103 cells/well)
Number of macrophages
Control
3x10^-5^ M
1x10^-4^ M
3x10^-4^ M
1x10^-9^ M
1x10^-8^ M
1x10^-3^ M
1x10^-7^ M
1x10^-6^ M
Dexamethasone Lidocaine
